# Snakebite in bedroom kills a physician in Cameroon: a case report

**DOI:** 10.11604/pamj.2016.24.231.7576

**Published:** 2016-07-13

**Authors:** Armand Nkwescheu, Leopold Cyriaque Donfack Mbasso, Franky Baonga Ba Pouth, Anastase Dzudie, Serge Clotaire Billong, Hermann Ngouakam, Joseph Le Doux Diffo, Hanny Eyongorock, Wilfred Mbacham

**Affiliations:** 1 Cameroon Society of Epidemiology, CaSE, Cameroon; 2 Faculty of Medicine and Biomedical Science, University of Yaoundé I, Cameroon; 3 Division of Health Operations Research, Ministry of Public Health of Cameroon, Cameroon; 4 Laboratory of Public Health for Research Biotechnologies, Biotechnology Center, UY I, Cameroon; 5 Expanded Program of Immunization, Ministry of Public Health of Cameroon, Cameroon; 6 Central Africa Field Epidemiology and Laboratory Training Program (CAFELTP), Cameroon; 7 Centre Régional de Prévention et la Lutte contre les Epidémies pour le Centre (CERPLE- C), Cameroon; 8 Department of Medicine, Douala General Hospital, Douala, Cameroon; 9 National AIDS Control Committee of Cameroon, Central Technical Group, Cameroon; 10 Synergies Africaines pour les Souffrances contre le SIDA, Yaoundé, Cameroon; 11 Cameroon Herpetology, Conservation Biology Foundation (CAMHERP-CBF), Cameroon; 12 Global Viral Cameroon (GVC), Cameroon; 13 National Medical Council of Pharmacists of Cameroon(ONPC), Cameroon; 14 Directorate of Pharmacy, Medicines and Laboratory, Ministry of Public Health of Cameroon, Cameroon

**Keywords:** Fatal snakebite, cobra, physician, Cameroon

## Abstract

The World Health Organization (WHO) classifies snake bites as neglected public health problem affecting mostly tropical and subtropical countries. In Africa there are an estimated 1 million snake bites annually with about half needing a specific treatment. Women, children and farmers in poor rural communities in developing countries are the most affected. Case management of snake bites are not adequate in many health facilities in developing countries where personnel are not always abreast with the new developments in snake bite management and in addition, quite often the anti-venom serum is lacking. We report the case of a medical doctor bitten by a cobra in the rural area of Poli, Cameroon while asleep in his bedroom. Lack of facilities coupled with poor case management resulted in a fatal outcome.

## Introduction

Snake bite is a neglected public health issue with about 5 million cases per year worldwide [1–3]. Among the cases, about half are true envenoming and up to 100.000 victims may have a fatal outcome [1]. The outcome of a snake bite depends on several factors, including the species of snake, the area of the body bitten, the amount of venom injected, the previous health condition of the victim and the case management at the health facility. Acute kidney injury can results in 5-30% of snake bites [4, 5] and, the outcome can be fatal, especially in cases of poor medical intervention. In sub-Saharan Africa, lack of anti-venom serum [6] and sometimes unscrupulous marketing of anti-venoms [7, 8] are contributing factors for a poor case- management of snake bites coupled with an inadequate Health systems [1] and often ill-prepared health professionals for the management of such medical emergency contributes to worsen the situation. We describe a fatal case of snake bite of a young medical officer in a rural area of Cameroon.

## Patient and observation

**Figure 1:**
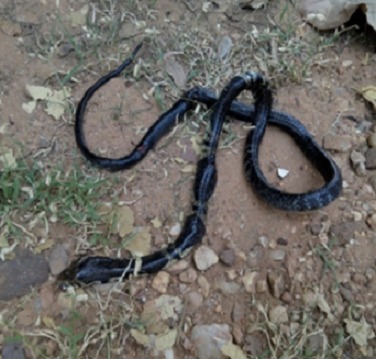
The snake which bit the physician in the bedroom

Dr F., aged 28 was a medical officer working with a Hospital in Poli (Latitude: 8°28' 59'' North; longitude: 13^°^15' 00'' East; Altitude: 461m above sea level) in the Northern region of Cameroon. He was sleeping in his bedroom when he awoke by a pain in the right hand. He drove to the emergency room of the district hospital on November 4, 2015 around 2:00AM by which time there was a swelling on his right hand. On arrival, he got the nurse on duty who confirmed the snake bite just 3-4cm below the right wrist. On clinical examination, he was conscious, anxious, pale and with a remarkable swelling of his right hand. His heart rate was 110 beats per minute and the blood pressure was 110/60 mm of Hg. The rest of the physical examination was unremarkable. No laboratory investigation was performed. The victim instructed the procedure for his case management that included a vein route drip of 500cc of ringer lactate, the administration of an anti-venom serum (1 vial in 500ml of 10% Dextrose); 8mg (2 vials) of dexamethasone IV; Anti-tetanus serum 1500 IU subcutaneously; Atropine 1 vial IV; Ranitidine 50mg 1 vial IV and paracetamol injectable 50mg IV. Two hours later, the patient presented with rigors, foaming at the mouth and difficulties of speech that followed by the complete loss of speech. The Patient was transferred to the Garoua reference hospital, located about 150 km from Poli, on a partial bitumen and earth road with many broken bridges. During the evacuation, the patient went into severe respiratory distress and finally a cardiac arrest. Resuscitation was unsuccessful and he died at about 40 km from Garoua. The snake was killed by the night watch and identified as a cobra of the Elapidae family, *Naja melanoleuca* (Figure 1).

## Discussion

Snake bite is a frequent health issue in the Health district of Poli. In 2014, 14cases of snake bites were recorded at the district hospital of whom 3 died. Amongst the 14with snake bite three did not receive anti-venom serum because it was not available at the time needed. One of the 3 patients died. Our case report is relevant for two reasons: 1) first the victim was the most skilled care provider in the area where shortage of medical staff is observed and was the person that guided his case management despite the stressful situation; 2) the rapid fatal outcome highlights the need to urgently address the issue of vulnerability of health personnel in such circumstances. Among the several factors, that could have influenced the fatal outcome of the case, the following issues deserve particular attention. The medical doctor was bitten by a cobra, a venomous and aggressive species [3]. He drove to the emergency room himself, therefore mobilizing skeletal muscles and increasing blood flow and circulation of the venom to vital organs. Pressure immobilization of the limb is recommended to contain venom within a bitten limb and delay its circulation through blood and the lymphatics to the nervous system and other vital organs. This preventive measure was not also taken. Anti-venom, the only effective treatment of envenomation was not administered in line with current recommendations [3]. Indeed, multiple injections would have been necessary to possibly rescue the situation. The anti-venom used is known not to be effective in Africa [7, 8]. The surroundings of the residence was bushy, an environmental risk factor [1]. Finally, it was not possible to anticipate a respiratory complication and provide assistance during the transfer of the patient to a higher reference level hospital. Moreover, the evacuation of the patient to the health facility was delayed for several hours because of the bad roads.

## Conclusion

The present case report demonstrates the need to update the capacity of rural health care facilities for the management of situations in enclaved areas. Important andurgent steps include providing high risk areas like Poli with sufficient stock of adequate anti-venom serum, adequate training of personnel on case management with timely and adequate evacuation facilities to a higher level health facility as the need arises.
